# Singular and combined effects of transcranial infrared laser stimulation and exposure therapy on pathological fear: a randomized clinical trial

**DOI:** 10.1017/S0033291721002270

**Published:** 2023-02

**Authors:** Eric D. Zaizar, Santiago Papini, F. Gonzalez-Lima, Michael J. Telch

**Affiliations:** 1 Department of Psychology, The University of Texas at Austin, Austin, TX, USA; 2 Institute for Mental Health Research, The University of Texas at Austin, Austin, TX, USA; 3 Institute for Neuroscience, The University of Texas at Austin, Austin, TX, USA; 4 Department of Psychiatry and Behavioral Sciences, Dell Medical School, The University of Texas at Austin, Austin, TX, USA

**Keywords:** Cytochrome-c-oxidase (CCO), dorsolateral prefrontal cortex (dlPFC), exposure therapy, non-invasive brain stimulation (NIBS), transcranial infrared laser stimulation (TILS), ventromedial prefrontal cortex (vmPFC)

## Abstract

**Background:**

Preclinical findings suggest that transcranial infrared laser stimulation (TILS) improves fear extinction learning and cognitive function by enhancing prefrontal cortex (PFC) oxygen metabolism. These findings prompted our investigation of treating pathological fear using this non-invasive stimulation approach either alone to the dorsolateral PFC (dlPFC), or to the ventromedial PFC (vmPFC) in combination with exposure therapy.

**Methods:**

Volunteers with pathological fear of either enclosed spaces, contamination, public speaking, or anxiety-related bodily sensations were recruited for this randomized, single-blind, sham-controlled trial with four arms: (a) Exposure + TILS_vmPFC (*n* = 29), (b) Exposure + sham TILS_vmPFC (*n* = 29), (c) TILS_dlPFC alone (*n* = 26), or (d) Sham TILS _dlPFC alone (*n* = 28). Post-treatment assessments occurred immediately following treatment. Follow-up assessments occurred 2 weeks after treatment.

**Results:**

A total of 112 participants were randomized [age range: 18–63 years; 96 females (85.71%)]. Significant interactions of Group × Time and Group × Context indicated differential treatment effects on retention (i.e. between time-points, averaged across contexts) and on generalization (i.e. between contexts, averaged across time-points), respectively. Among the monotherapies, TILS_dlPFC outperformed SHAM_dlPFC in the initial context, *b* = −13.44, 95% CI (−25.73 to −1.15), *p* = 0.03. Among the combined treatments, differences between EX + TILS_vmPFC and EX + SHAM_vmPFC were non-significant across all contrasts.

**Conclusions:**

TILS to the dlPFC, one of the PFC regions implicated in emotion regulation, resulted in a context-specific benefit as a monotherapy for reducing fear. Contrary to prediction, TILS to the vmPFC, a region implicated in fear extinction memory consolidation, did not enhance exposure therapy outcome.

## Introduction

Knowledge of the neural circuits underlying emotion dysregulation in anxiety and trauma-related disorders can be leveraged to identify cortical targets for non-invasive brain stimulation (NIBS), either as an adjunct to behavioral interventions or as a standalone treatment (Ressler & Mayberg, 2007; Vicario, Salehinejad, Felmingham, Martino, & Nitsche, 2019). Extinction is the emotional learning process underlying exposure therapy (Graham & Milad, 2011). The ventromedial prefrontal cortex (vmPFC) has been implicated in neural circuits for consolidation and retrieval of fear extinction memory in animal and human studies by consistent evidence, such as vmPFC lesions impairing extinction retrieval, vmPFC stimulation strengthening extinction memory, vmPFC metabolic and electrophysiological responses being potentiated by extinction, and vmPFC activation being correlated with extinction behavior (Barrett & Gonzalez-Lima, 2018; Gilmartin, Balderston, & Helmstetter, 2014; Quirk, Garcia, & Gonzalez-Lima, 2006; Quirk & Mueller, 2008). Preliminary transcranial direct current stimulation (tDCS) studies with humans show that stimulating the vmPFC improves fear extinction (Nuñez, Zinbarg, & Mittal, 2019). Additionally, targeting the dorsolateral prefrontal cortex (dlPFC) with either standalone tDCS or transcranial magnetic stimulation (TMS) has been shown to reduce anxiety disorder symptom severity (Vicario et al., 2019). Importantly, meta-analyses of TMS for anxiety and trauma-related disorders have shown medium to large treatment effects (Cirillo et al., 2019; Cui et al., 2019) and tDCS has shown large effect sizes for reducing core symptoms of post-traumatic stress disorder (PTSD), supporting the efficacy of NIBS (Kan, Zhang, Zhang, & Kranz, 2020).

Here we present the first clinical trial treating pathological fear with transcranial infrared laser stimulation (TILS), a mechanistically distinct form of NIBS that improves neuronal bioenergetics by stimulating cytochrome-c-oxidase (CCO) activity (Gonzalez-Lima & Barrett, 2014; Hamblin, 2016; Rojas & Gonzalez-Lima, 2013). By targeting CCO, a fundamental enzyme for oxidative energy production, TILS may provide some advantages over other forms of NIBS due to its more well-established mechanistic specificity (Gonzalez-Lima & Barrett, 2014). As brain physiology is critically dependent on oxygenation for energy production, the mechanistic details of TILS on CCO are tied to cognitive processing by the brain: (a) near-infrared photons penetrate into the cerebral cortex (Salehpour et al., 2019) and oxidize CCO because CCO is the major intracellular acceptor of photons from red-to-near-infrared light (Rojas & Gonzalez-Lima, 2013); (b) CCO catalyzes the reduction of oxygen to water that allows mitochondria to produce adenosine triphosphate (ATP), the energy source in cell metabolism (Gonzalez-Lima, Barksdale, & Rojas, 2014); and (c) CCO also catalyzes the synthesis of the vasodilator nitric oxide (NO) under low oxygen conditions (Poyton & Hendrickson, 2016). Therefore, TILS of the PFC augments CCO-catalyzed oxygen consumption, ATP production, and NO-mediated vasodilation, which constitute its downstream mechanism for augmenting brain activity. Direct measurement evidence for this mechanism is provided by 10 randomized sham-controlled brain activity studies showing that TILS influences human PFC activity by photo-oxidation of CCO and causes hemodynamic, electrophysiological, and functional connectivity effects in the default mode network that augment PFC-based cognitive brain functions (Holmes et al., 2019; Pruitt et al., 2020; Saucedo et al., 2021; Tian, Hase, Gonzalez-Lima, & Liu, 2016; Urquhart et al., 2020; Vargas et al., 2017; Wang, Dmochowski, Husain, Gonzalez-Lima, & Liu, 2017a; Wang et al., 2017b, 2018, 2019). Five randomized sham-controlled human cognitive studies also provide measurement evidence of TILS-induced beneficial effects on PFC-modulated attention, memory, and executive functions (Barrett & Gonzalez-Lima, 2013; Blanco, Maddox, & Gonzalez-Lima, 2017a; Blanco, Saucedo, & Gonzalez-Lima, 2017b; Disner, Beevers, & Gonzalez-Lima, 2016; Hwang, Castelli, & Gonzalez-Lima, 2016). Together these 15 human studies with over 500 subjects demonstrate that TILS to the forehead at 1064 nm wavelength engages PFC oxygenation that is tied to PFC-based cognitive functions by promoting PFC activity that modulates network electrophysiology. Our aim was to examine the potential of TILS as (a) an exposure therapy enhancer by targeting the vmPFC, which plays a critical role in fear extinction (Sevenster, Visser, & D'Hooge, 2018) and (b) a monotherapy by targeting the dlPFC, which has been implicated in emotion regulation (Buhle et al., 2013).

The application of fear extinction principles through repeated exposure to fear-provoking targets is a powerful intervention for reducing pathological fear (Milad & Quirk, 2012; Telch, Cobb, & Lancaster, 2014b). However, a substantial minority of patients experience a return of fear in new contexts or after the passage of time, highlighting the need for exposure-augmentation strategies (Vervliet, Craske, & Hermans, 2013). Although pharmacologic approaches have predominated the exposure-augmentation research, NIBS is a compelling alternative that can selectively target cortical regions with putative roles in extinction learning (Rojas & Gonzalez-Lima, 2013; Sathappan, Luber, & Lisanby, 2019). Extinction enhancement with methylene blue (MB), a pharmacological agent sharing the same mechanistic CCO target as TILS, has been successfully translated from rodent models to exposure therapy for claustrophobia (Telch et al., 2014a) and PTSD (Zoellner et al., 2017). However, prior work with TILS and extinction is limited to rodents, where post-extinction TILS improved fear extinction memory retention by boosting CCO in the PFC (Rojas, Bruchey, & Gonzalez-Lima, 2012). Therefore, clinical trials of the effect of vmPFC-TILS on exposure therapy are warranted.

Another promising NIBS target is the dlPFC, which is implicated in the cognitive control of emotion (Buhle et al., 2013) and is deficiently recruited in pathological fear (Zilverstand, Parvaz, & Goldstein, 2017). While dlPFC-TILS facilitated sustained attention (Barrett & Gonzalez-Lima, 2013), executive function (Barrett & Gonzalez-Lima, 2013; Blanco et al., 2017b), and mood (Barrett & Gonzalez-Lima, 2013) in healthy subjects and enhanced attention bias modification treatment for sub-clinically depressed individuals (Disner et al., 2016), evidence for the therapeutic effect of dlPFC-TILS for anxiety is limited to one uncontrolled study (Schiffer et al., 2009). This warrants further testing of the therapeutic potential of dlPFC-TILS as a monotherapy for pathological fear.

Drawing from these findings, we conducted a four-arm randomized placebo-controlled trial aimed at addressing two primary questions: (a) Does post-session administration of TILS targeting the vmPFC – a cortical region strongly implicated in fear extinction, enhance exposure therapy outcomes relative to sham TILS? (b) Does TILS monotherapy targeting the dlPFC – a cortical region implicated in emotion regulation, outperform sham stimulation in reducing naturally acquired pathological fear? Lastly, to test whether subjects in the higher clinical range benefit more from either exposure therapy, TILS, or their combination, we also examined baseline symptom severity as a moderator of treatment outcome.

## Methods

### Participants

Treatment-seeking volunteers (*N* = 112) with pathological fear of enclosed spaces, contamination, public speaking, or bodily sensations were recruited from the university's student population and greater Austin community. Inclusion criteria were (1) aged 18–65 years; and (2) peak-fear rating of 50 or higher on two behavioral assessments (see measures). The minimum peak-fear criterion was selected to ensure that only participants displaying sufficient fear during the behavioral assessments were included in the trial. Exclusion criteria were (1) suicide risk; (2) concurrent exposure-based treatment; (3) medication change within 6 weeks; and (4) contraindicated medical or neurological condition. Written informed consent was collected prior to procedures, which were approved by the Institutional Review Board of The University of Texas at Austin. The complete protocol is provided in online Supplementary Material 1 and is described in our methods publication (Zaizar, Gonzalez-Lima, & Telch, 2018).

### Procedures

#### Recruitment

**Fig. 1.**
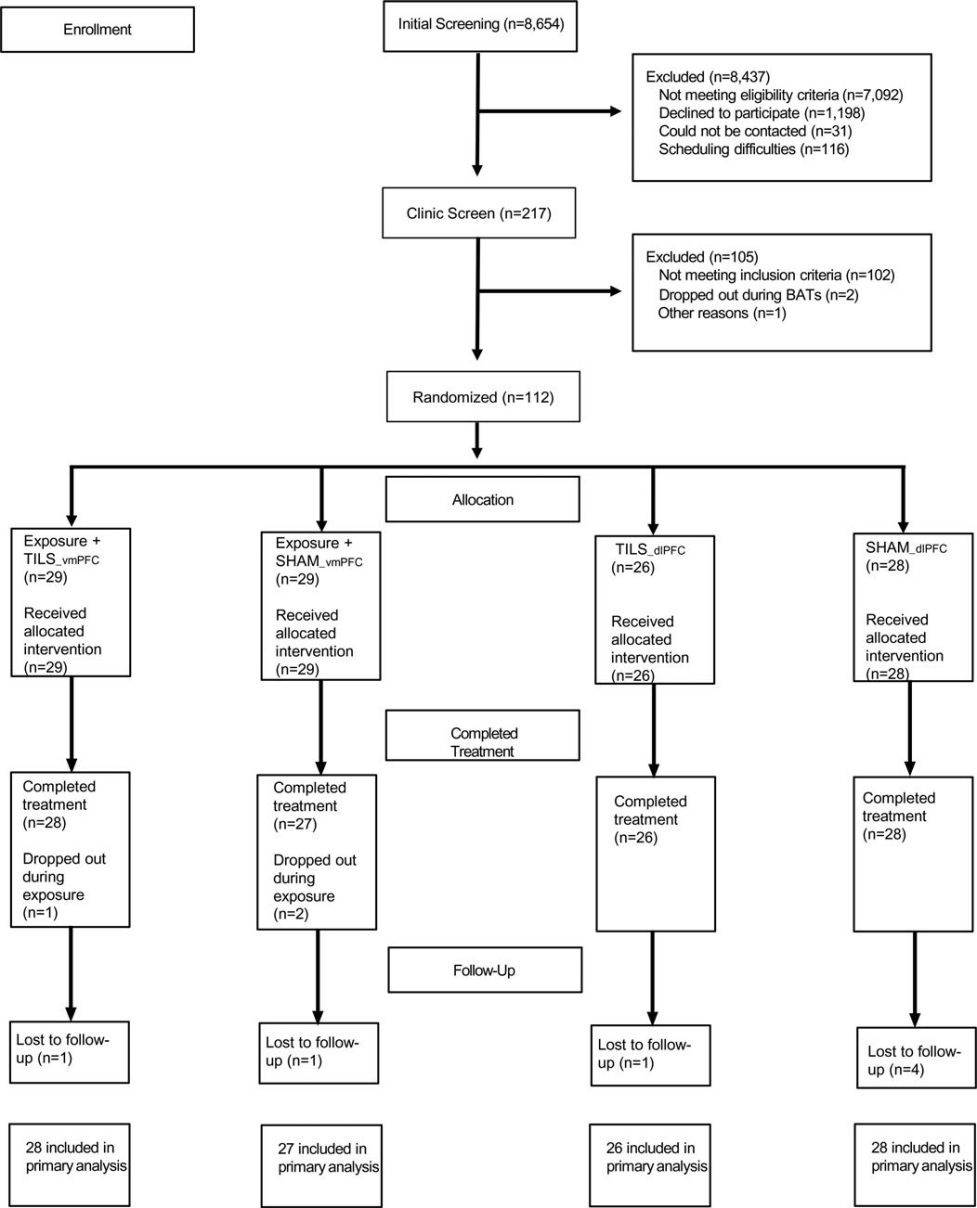
CONSORT flow diagram.

Figure 1 summarizes participant flow through the study from October 2014 to March 2020. An online prescreen was used to assess initial eligibility and recruit individuals who showed elevated scores (2 s.d.s above mean) on domain-specific validated symptom inventories: Claustrophobia Questionnaire (Radomsky, Rachman, Thordarson, McIsaac, & Teachman, 2001), Obsessive-Compulsive Inventory-Revised (Foa et al., 2002), Liebowitz Social Anxiety Scale Self-Report (Heimberg et al., 1999), or Anxiety Sensitivity Index-3 (Taylor et al., 2007). Participants were enrolled by research assistants who conducted the baseline assessment.

#### Randomization

The randomization sequence was generated by the first author (EDZ). Eligible participants were stratified on fear domain, sex, and pretreatment peak-fear and block randomized to one of four treatment arms: (a) Exposure to feared targets + TILS of the vmPFC (EX + TILS_vmPFC); (b) Exposure to feared targets + sham TILS of the vmPFC (EX + SHAM_vmPFC); (c) TILS of the dlPFC (TILS_dlPFC); and (d) Sham TILS of the dlPFC (SHAM_dlPFC). Participants were blind as to whether they received active or sham TILS in the exposure therapy arms and the monotherapy arms.

### Interventions

#### Transcranial infrared laser stimulation (TILS)

Prior to laser stimulation, all participants viewed a video demonstration of the brain stimulation procedures which included treatment rationales highlighting improved emotion regulation in the two monotherapy conditions (TILS_dlPFC/SHAM_dlPFC) or fear extinction enhancement in the two stimulation plus exposure therapy conditions (EX + TILS_vmPFC/EX + SHAM_vmPFC).

In the two monotherapy conditions, TILS was administered bilaterally to the dlPFC (F3 and F4 on the electroencephalographic International 10–20 System) for 60 s in four alternating cycles for 8 min total with a 1064 nm wavelength Model CG-5000 laser diode supplied by Cell Gen Therapeutics, LLC (HD Laser Center, Dallas, TX, USA). We have shown in a published video our laser device on the head with stimulation targets and laser stimulation procedure and setup (Zaizar et al., 2018). We used laser parameters and standard operating procedures reviewed and approved by the Laser Safety Office of the University of Texas at Austin and published previously (Zaizar et al., 2018). The laser had a collimated beam circular area of 13.6 cm^2^. The measured laser power output was 3.4 W. Each forehead area treated received a power density (irradiance) of 0.25 W/cm^2^ (3.4 W/13.6 cm^2^). For the 8 min stimulation, the total laser energy delivered was 1632 J (3.4W × 480 s). The laser energy density (fluence) was 120 J/cm^2^ (0.25 W/cm^2^ × 480 s). SHAM administration followed the same 8 min procedure but delivered only 5 s of laser stimulation per cycle. Thus, the SHAM condition received 3.4 W × 5 s equal to 17 J for a total energy density of 0.25 W/cm^2^ × 5 s equal to 1.25 J/cm^2^ (i.e. less than 1% of the energy density used in the active laser groups). This 5 s stimulation is sufficient to provide a brief sensation of slight heat (as active placebo) at the onset of each 1 min cycle, using a small fraction of the energy received by the experimental groups, but insufficient to influence behavioral or brain function (Barrett & Gonzalez-Lima, 2013; Saucedo et al., 2021). Parameters set for our TILS and SHAM stimulation procedures were identical to our previously published studies (Barrett & Gonzalez-Lima, 2013; Blanco et al., 2017b; Disner et al., 2016; Hwang et al., 2016). We elected to use an active placebo comparator based on their enhanced utility to reduce the risk of participant unblinding relative to traditional placebos (Jensen, Bielefeldt, & Hróbjartsson, 2017).

In the two exposure therapy arms, TILS or Sham stimulation was administered immediately following the completion of exposure therapy and consisted of bilateral stimulation to the vmPFC (FP1 and FP2, International 10–20 System for EEG) for 60 s in four alternating cycles for 8 min total (EX + TILS_vmPFC) or the same procedure (EX + SHAM_vmPFC) for 5 s of laser stimulation per cycle.

Participants were fitted with protective eyewear and were asked to close their eyes for the duration of this procedure. This ensured eye safety and aided in keeping participants blind as to whether they received TILS or SHAM. Furthermore, as a manipulation check, we measured whether participants believed they received active or sham stimulation as well as their confidence in this belief.

#### Exposure therapy

Participants randomized to EX + TILS_vmPFC and EX + SHAM_vmPFC completed single-session manualized exposure therapy protocols consisting of repeated trials confronting one of four phobic targets: (a) claustrophobia (lying inside a tightly enclosed chamber) (Telch et al., 2014a); (b) contamination fear (touching a mixture of dirt, dead insects, and hair) (Cougle, Wolitzky-Taylor, Lee, & Telch, 2007); (c) public speaking fear (giving a speech in front of a live audience) (Smits, Powers, Buxkamper, & Telch, 2006); and (d) fear of benign somatic sensations (repeated inhalations of a gas mixture of 35% CO_2_/65% O_2_) (Telch, Rosenfield, Lee, & Pai, 2012).

### Measures

#### Primary outcome

**Fig. 2.**
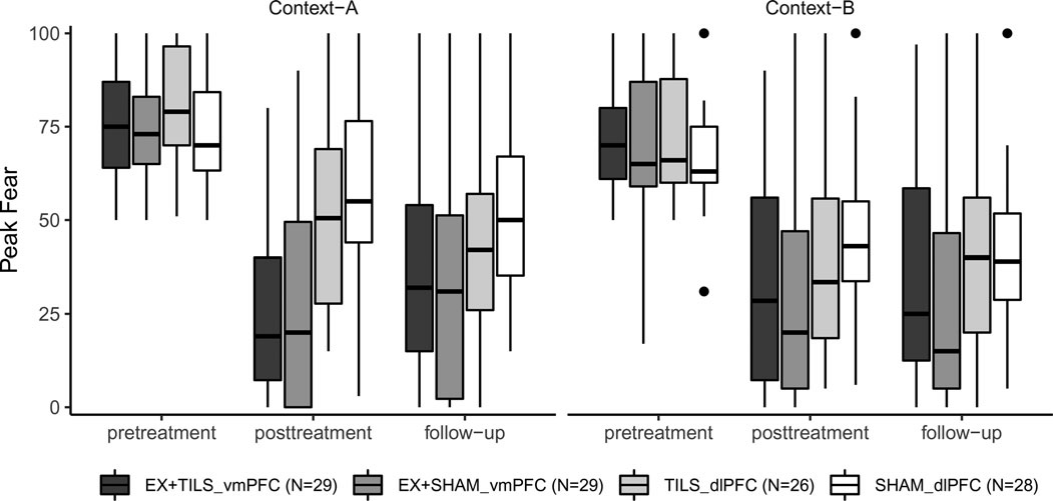
Boxplot of peak-fear levels in the behavioral approach tasks. *Note*: Boxplots illustrate median and distribution (interquartile range) of raw data values.

The primary outcome was self-reported peak-fear (range: 0–100) measured at the termination of two behavioral approach tests (BATs; Context A; Context B) each conducted at pre-randomization, post-TILS, and at 2-week follow-up (Fig. 2). Assessing the outcome in two contexts allowed us to examine the generalization of treatment effects across two distinct contexts. Note that across groups, the context effect may also involve an order effect because Context A always preceded Context B. In the exposure groups, this effect also captured differences between testing in treatment and non-treatment contexts because Context A used the exposure therapy stimuli and Context B used novel stimuli. Stimuli used for the BAT contexts were specific to each fear domain and are outlined in Table 1. Immediately following each BAT context, subjects rated the maximum level of fear (peak-fear: 0–100) they experienced during the BAT. All BATs lasted up to 15 s (except for the 10 s 35% CO_2_ challenge and 1 min respiratory task, see Table 1). This method for indexing fear responses is based on prior research (Cougle et al., 2007; Smits et al., 2006; Telch et al., 2012, 2014a).

**Table 1.** Summary of behavioral approach test contexts for each fear domain

Domain	Context A	Context B
Fear of enclosed spaces	Lying supine within a tightly enclosed wooden chamber	Lying supine within a mock fMRI scanner
Fear of contamination	Touching a mixture of dirt, dead insects, and hair, with both hands	Touching a toilet with potting soil smeared on the inside, with both hands
Fear of public speaking	Speaking on a topic selected for treatment, 3 people observing, not videotaped	Speaking on a new topic (not practiced during exposure), via skype to a team of 3 speech evaluators, and videotaped
Anxiety sensitivity	Inhaling 35% CO_2_/65% O_2_ gas mixture for 10 s	1 min voluntary respiratory challenge

*Note*: Context A stimulus was also encountered during exposure therapy.

#### Treatment moderator

Pre-randomization peak-fear ratings served as treatment moderators (see analyses section).

### Statistical analysis

#### Sample size calculation

Prior clinical trials from our group have examined the impact of MB, a pharmacologic intervention that shares the same CCO mechanism as TILS, on the treatment of claustrophobia (Telch et al., 2014a) and PTSD (Zoellner et al., 2017). These studies found evidence of enhancing effects in samples of 19–23 subjects per group. For this pilot trial of TILS as a singular or combined treatment for pathological fear, our aim was to recruit 30 participants per group.

#### Primary outcome

Analyses were conducted in R (version 3.6.1) and applied a two-tailed significance threshold of *α* = 0.05. The primary outcome of peak-fear in the BATs was analyzed in a mixed model with Group (EX + TILS_vmPFC, EX + SHAM_vmPFC, TILS_dlPFC, SHAM_dlPFC), Time (Post-treatment, Follow-up), Context (A, B), their interactions, and a random subject effect. The effect of Baseline-Fear was included to adjust for pre-randomization levels of the outcome and its interaction with Group was included to test its moderating impact on treatment effects. Pairwise comparisons of significant interaction effects were used to test differential treatment effects on *retention* (i.e. Group × Time) and *contextual generalization* (i.e. Group × Context). Here the retention interaction answers the question ‘Are there group differences in the retention of treatment effects from post-treatment to follow-up?’ and the context interaction answers the question ‘Are there group differences in how treatment effects generalize across distinct contexts?’.

The trial protocol paper (Zaizar et al., 2018) originally proposed measuring the primary outcome in a 2 × 2 ANCOVA of Exposure Therapy (yes or no) and TILS (yes or no). However, while data collection was ongoing and prior to analyses being conducted, the decision was made to analyze the four groups separately because vmPFC-TILS and dlPFC-TILS represent two distinct treatment approaches given the difference in stimulation site. In order to include all participants, consistent with the principles of intent-to-treat analyses, we used mixed models (which applies robust maximum likelihood estimation to estimate the effect of missing data) in place of ANCOVA (which applies listwise deletion of any participants of missing data).

#### Treatment moderator

Preliminary analyses indicated the primary outcome model described above fits the data better than a less complex model without Group × Baseline-Fear, χ^2^(3) = 16.87, *p* < 0.001. Therefore, the moderating impact of baseline severity was tested within the primary outcome model. The protocol paper (Zaizar et al., 2018) proposed additional potential moderation analyses (e.g. moderation by fear domain type). However, given the sample size for this pilot project, we decided to limit analyses to the primary outcome and moderation by baseline fear.

## Results

### Sample characteristics

Table 2 presents baseline demographic and clinical characteristics of the sample. Fear domains were distributed across enclosed spaces [*n* = 36 (32.14%)), bodily sensations (*n* = 32, (28.57%)], contamination [*n* = 22, (19.64%)], or public speaking [*n* = 22, (19.64%)]. Domain-specific validated measures were in the pathological range based on cutoffs and normative data (Foa et al., 2002; Heimberg et al., 1999; Radomsky et al., 2001; Taylor et al., 2007).

**Table 2.** Demographic and clinical characteristics of treatment groups

Characteristic	EX + TILS_vmPFC *n* = 29	EX + SHAM_vmPFC *n* = 29	TILS_dlPFC *n* = 26	SHAM_dlPFC *n* = 28
Age, mean (s.d.), y	20.55 (5.35)	19.76 (2.67)	19.00 (1.13)	22.57 (10.85)
Female sex	24 (82.76)	25 (86.21)	23 (88.46)	24 (85.71)
Self-reported race or ethnicity				
American Indian/Alaska Native	0	0	1 (3.85)	0
Asian	8 (27.59)	6 (20.69)	3 (11.54)	7 (25.00)
Black/African American	1 (3.45)	1 (3.45)	2 (7.69)	2 (7.14)
White (not Hispanic or Latino)	8 (27.59)	14 (48.28)	10 (38.46)	9 (32.14)
Hispanic or Latino	12 (41.38)	8 (27.59)	10 (38.46)	10 (35.71)
Full time student	22 (75.86)	20 (68.97)	22 (84.62)	19 (67.86)
Employed	7 (24.14)	10 (34.48)	9 (34.62)	5 (17.86)
Fear domain and severity				
Bodily sensations	8 (27.59)	8 (27.59)	7 (26.92)	9 (32.14)
ASI-3 total, M (s.d.)	57.25 (7.83)	45.00 (14.95)	54.29 (9.05)	47.89 (11.07)
Closed spaces	10 (34.48)	9 (31.03)	9 (34.62)	8 (28.57)
CLQ total, M (s.d.)	41.40 (14.78)	34.00 (9.27)	51.89 (9.45)	39.88 (9.01)
Contamination	5 (17.24)	6 (20.69)	5 (19.23)	6 (21.43)
OCI-R, M (s.d.)	41.00 (9.70)	39.33 (9.29)	28.60 (16.27)	34.17 (14.47)
Public speaking	6 (20.69)	6 (20.69)	5 (19.23)	5 (17.86)
LSAS total, M (s.d.)	94.67 (19.17)	88.67 (25.44)	71.40 (32.53)	92.20 (17.05)
CEQ, treatment credibility, M (s.d.)	4.87 (1.43)	5.62 (1.61)	4.21 (1.64)	5.25 (1.64)
CEQ, treatment expectancy, M (s.d.)	31.72 (16.92)	37.59 (22.47)	27.69 (19.25)	44.64 (21.68)

ASI-3, Anxiety Sensitivity Index 3; CLQ, Claustrophobia Questionnaire; OCI-R, Obsessive-Compulsive Inventory-Revised; LSAS, Liebowitz Social Anxiety Scale; CEQ, Credibility/Expectancy Questionnaire.

### Treatment integrity, credibility, safety

Dropout proportions were not significantly different across groups, [EX + TILS_vmPFC 1 (3.45%); EX + SHAM_vmPFC 2 (6.90%); TILS_dlPFC 0; SHAM_dlPFC 0; Fisher's exact test, *p* = 0.62], and no adverse events were reported. Group differences in the proportion of participants who believed they received active TILS were non-significant [EX + TILS_vmPFC: 20 (71.43%); EX + SHAM_vmPFC: 20 (74.07%); TILS_dlPFC: 19 (73.08%); SHAM_dlPFC: 19 (67.86%); *p* = 0.96]. Unexpectedly, SHAM_dlPFC had *higher* mean treatment credibility and expectancy ratings than EX + SHAM_vmPFC and TILS_dlPFC (Table 2). Together, this suggests SHAM stimulation functioned as a rigorous active placebo control condition: across both SHAM groups, most participants believed they received TILS, and treatment expectancy and credibility was higher than or comparable to the groups that included active TILS.

### Primary outcome

#### Omnibus test

Figure 2 shows raw peak-fear values across time-points and contexts; full model statistics are provided in the online Supplementary Materials. Although the Group × Time × Context interaction was non-significant (*p* = 0.16), significant interactions of Group × Time (*p* = 0.02) and Group × Context (*p* = 0.02) indicated differential treatment effects on retention (i.e. between time-points, averaged across contexts) and on generalization (i.e. between contexts, averaged across time).

#### Retention effects

Across the two post-treatment time-points, EX + TILS_vmPFC did not outperform EX + SHAM_vmPFC (both *p*s > 0.36), and TILS_dlPFC did not outperform SHAM_dlPFC (both *p*s > 0.09). However, both exposure groups had lower fear levels relative to the non-exposure groups [EX + TILS_vmPFC *v.* TILS_dlPFC: *b* = −13.30, 95% CI (−25.45 to −1.15), *p* = 0.03; EX + TILS_vmPFC *v.* SHAM_dlPFC: *b* = −23.87, 95% CI (−35.7 to −11.95), *p* < 0.001; EX + SHAM_vmPFC *v.* TILS_dlPFC: *b* = −15.88, 95% CI (−28.13 to −3.63), *p* = 0.01; EX + SHAM_vmPFC *v.* SHAM_dlPFC: *b* = −26.44, 95% CI (−38.47 to −14.41), *p* < 0.001]. But, at follow-up, the two exposure groups significantly outperformed SHAM_dlPFC only [EX + TILS_vmPFC *v.* SHAM_dlPFC: *b* = −13.87, 95% CI (−25.98 to −1.76), *p* = 0.03; EX + SHAM_vmPFC *v.* SHAM_dlPFC: *b* = −19.55, 95% CI (−31.78 to −7.32), *p* = 0.002]. All other comparisons were non-significant.

#### Contextual generalization effects

Differences between EX + TILS_vmPFC and EX + SHAM_vmPFC were non-significant in each context (both *p*s > 0.31), suggesting that the addition of TILS_vmPFC did not enhance contextual generalization of exposure effects. However, TILS_dlPFC outperformed SHAM_dlPFC in assessment context A [TILS_dlPFC *v.* SHAM_dlPFC: *b* = −13.44, 95% CI (−25.73 to −1.15), *p* = 0.03], providing some evidence of the impact of dlPFC-TILS on fear reduction in the initial testing context. Additionally, exposure groups had lower fear relative to SHAM_dlPFC in assessment context A [EX + TILS_vmPFC *v.* SHAM_dlPFC: *b* = −24.91, 95% CI (−36.92 to −12.9), *p* < 0.001; EX + SHAM_vmPFC *v.* SHAM_dlPFC: *b* = −26.87, 95% CI (−38.96 to −14.78), *p* < 0.001] and in assessment context B [EX + TILS_vmPFC *v.* SHAM_dlPFC: *b* = −12.83, 95% CI (−24.88 to −0.78), *p* = 0.04; EX + SHAM_vmPFC *v.* SHAM_dlPFC: *b* = −19.12, 95% CI (−31.33 to −6.91), *p* = 0.003]. But only EX + SHAM_vmPFC outperformed TILS_dlPFC (Context A: EX + SHAM_vmPFC *v.* TILS_dlPFC: *b* = −13.42, 95% CI (−25.77 to −1.07), *p* = 0.04; Context B: EX + SHAM_vmPFC *v.* TILS_dlPFC: *b* = −12.68, 95% CI (−24.9 to −0.37), *p* = 0.046]. All other comparisons were non-significant.

### Moderation by baseline-fear

**Fig. 3.**
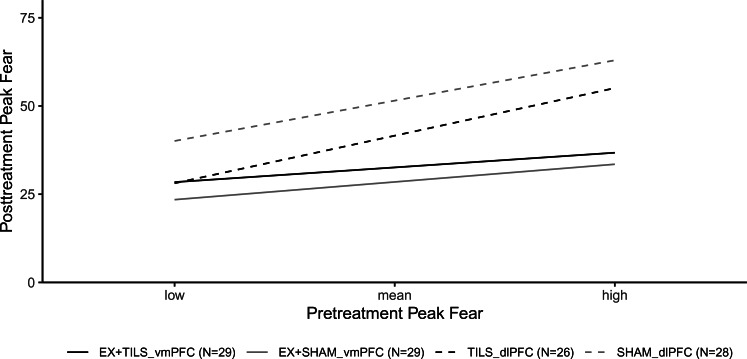
Model-based estimates of moderation effects of the primary outcome by pretreatment peak-fear levels. *Note*: The Group × Baseline-Fear interaction (averaged across time-points and contexts) was significant, *p* < 0.001.

Figure 3 shows the significant Group × Baseline-Fear interaction (averaged across time-points and contexts), *p* < 0.001. At low levels of baseline-fear, the only significant difference was between EX + SHAM_vmPFC and SHAM_dlPFC [*b* = −16.63, 95% CI (−29.14 to −4.13), *p* = 0.01], whereas at high levels of baseline-fear, the two exposure conditions outperformed each of the monotherapy conditions [EX + TILS_vmpFC *v.* TILS_dlPFC: *b* = −18.31, 95% CI (−30.95 to −5.67), *p* = 0.01; EX + TILS_vmPFC *v.* SHAM_dlPFC: *b* = −26.19, 95% CI (−39.08 to −13.29), *p* < 0.001; EX + SHAM_vmPFC *v.* TILS_dlPFC: *b* = −21.58, 95% CI (−34.42 to −8.75), *p* < 0.001; EX + SHAM_vmPFC *v.* SHAM_dlPFC: *b* = −29.46, 95% CI (−42.53 to −16.39), *p* < 0.001].

## Discussion

This was the first rigorous test of the singular and combined effects of a single administration of TILS and single-session exposure therapy for reducing naturally acquired pathological fear. Internal validity was maximized by utilizing active sham stimulation as a comparator and by measuring the success of this manipulation. Most subjects (69.64%) reported believing that they received real TILS and there were no significant group differences with respect to this belief. External validity was strengthened by including participants with a diverse range of pathological fears.

We did not find evidence that vmPFC-TILS enhanced the retention or contextual generalization of fear extinction learning. Given that *systemic* engagement of the same mechanistic target (i.e. neuronal bioenergetics) with oral administration of MB (Gonzalez-Lima & Bruchey, 2004; Wrubel, Barrett, Shumake, Johnson, & Gonzalez-Lima, 2007) effectively augmented exposure therapy (Telch et al., 2014a; Zoellner et al., 2017), targeting the vmPFC with TILS may have failed to influence the extensive network of brain regions involved in post-extinction memory consolidation (Barrett, Shumake, Jones, & Gonzalez-Lima, 2003; Sevenster et al., 2018). Though photobiomodulation improved fear extinction in rodents (Rojas et al., 2012), the stimulation included all the rodent brain relative to only the vmPFC in humans (Carlén, 2017; Fullana et al., 2018), that may have made it more feasible to engage the fear extinction neuronal network more completely. Further research is necessary to identify optimal targets for enhancing exposure therapy with TILS using brain imaging to assess the specificity of target engagement (Wang et al., 2017b). Whether the posterior part of the vmPFC was sufficiently engaged by TILS is unclear because the existing human studies documenting the effects of TILS on CCO and/or hemodynamics have measured from the anterior vmPFC at FP1/FP2 points or the anterior dlPFC at F3/F4 points (Holmes et al., 2019; Pruitt et al., 2020; Saucedo et al., 2021; Tian et al., 2016; Wang et al., 2017b, 2018). However, recent simulations of TILS penetration into the human head have shown that 1064 nm photons reach deep into the white matter posterior to the vmPFC, although with much reduced power levels than to anterior PFC regions (Huang, Kao, Sung, & Abraham, 2020; Tian, Varghese, Tran, Fang, & Gonzalez-Lima, 2020). An intranasal stimulation approach will not likely improve penetration into the mPFC because intranasal lasers have a very small beam area, which results in significantly less penetration than the larger laser beam disk areas that can be used in the forehead (Huang et al., 2020; Tian et al., 2020). Additionally, extensive evidence supports the role of the vmPFC in both retrieval and consolidation of extinction (Quirk & Mueller, 2008). Therefore, an alternate timing of TILS to the vmPFC could be before the retrieval test, but this timing would have more limited potential in the clinical practice of exposure therapy.

In contrast, we found weak-to-moderate support for the use of bilateral dlPFC-TILS monotherapy for reducing fear. Specifically, dlPFC-TILS attenuated fear in the first two assessment contexts, even though participants did not receive exposure therapy. It is important to note that mean peak-fear levels were lower in the second assessment context relative to the first across both monotherapy conditions (Fig. 2). Given that Context A was more fear provoking, it is plausible that Context A may have provided a more sensitive test of treatment effects than Context B. Our suggestive finding is consistent with research identifying the dlPFC as a stimulation target for improving emotion regulation (Buhle et al., 2013; Zilverstand et al., 2017). However, assessment of the therapeutic potential of dlPFC-TILS should take into account that EX + SHAM_vmPFC was associated with even greater fear reduction, highlighting the clinically meaningful potency of a single session of exposure therapy. Additionally, the exposure groups were more effective for individuals with high baseline-fear, on average across time-points and contexts. This is consistent with the already well-established efficacy of exposure therapy for clinical fears (Foa & McLean, 2015; Powers, Halpern, Ferenschak, Gillihan, & Foa, 2010) and underscores the importance of comparing novel interventions to this goal standard approach while considering pretreatment severity. Indeed, exposure therapy may be indicated for those in the higher phobic range. Nevertheless, our tentative finding can guide future work with dlPFC-TILS for pathological fear, which should examine increased doses (i.e. repeated stimulation) and lateralizing stimulation to the right dlPFC based on other NIBS studies (Vicario et al., 2019) and recent imaging work demonstrating that the right dlPFC is a major hub for outgoing connections to other brain regions (Li et al., 2018).

Given that we did not find support for vmPFC-TILS as an exposure therapy enhancer but did observe some therapeutic benefit of dlPFC-TILS as a monotherapy, this begs the question as to whether combining exposure therapy with dlPFC-TILS may serve as a promising augmentation treatment approach for future work. Relatedly, a recent meta-analysis highlighted the involvement of the dlPFC in human fear extinction learning (Fullana et al., 2018), consistent with neural models emphasizing higher cognitive circuits in human fear processes (LeDoux & Pine, 2016). Of note, one fMRI study observed increased right dlPFC activity and decreased amygdala activity immediately after a single session of exposure therapy (Hauner, Mineka, Voss, & Paller, 2012). However, at 6-month follow-up, only attenuated amygdala activity was observed. These findings suggest that the right dlPFC may have a ‘time-limited’ role in successful therapeutic learning and provide guidance for timing cortical stimulation to potentially enhance exposure therapy. Therefore, future studies should also test the effects of dlPFC-TILS on exposure therapy by specifically targeting the right dlPFC immediately after exposure.

### Limitations

Our design limits inferences (a) beyond a *single* administration of both interventions either alone or in combination; (b) regarding the effects of vmPFC stimulation *outside* of the context of exposure; or (c) regarding the effects of dlPFC stimulation *within* the context of exposure. This would have required a fully crossed six-arm design which was not feasible given our resources for this pilot. Second, our sample size precluded investigation of additional treatment moderation effects (e.g. fear domain type). Third, the relatively young age of the subjects may play a role in the TILS effects, as a new study comparing young and older subjects showed that there was a greater TILS effect on CCO with increasing age, while TILS effects on hemodynamics decreased with increasing age (Saucedo et al., 2021).

## Conclusions

The field of NIBS for pathological fear is still in its infancy, especially with respect to combining this approach with exposure therapy. This pilot trial represents the first attempt to characterize the clinical benefits of TILS alone and in concert with extinction-based therapy for pathological fear. Although we did not find evidence of improved exposure outcomes after vmPFC-TILS, our trial partially demonstrated that anxiolytic effects may be achieved through dlPFC-TILS. Given that this research is in an early phase and that we found only limited support for dlPFC-TILS as a monotherapy, it is important to interpret these initial findings cautiously. Subsequent investigations with this approach are needed to: (a) optimize dosing strategies for vmPFC-TILS or test alternative cortical targets for augmenting exposure therapy with TILS, and (b) evaluate the benefit of repeated and/or lateralized dlPFC-TILS as a monotherapy for pathological fear, similarly to TILS studies done for other purposes that used five sessions (O'Donnell, Barrett, Fink, Garcia-Pittman, & Gonzalez-Lima, 2021; Vargas et al., 2017).

## Supplementary material

For supplementary material accompanying this paper visit https://doi.org/10.1017/S0033291721002270. click here to view supplementary material
