# Supplementary material for: Singular and combined effects of transcranial infrared laser stimulation and exposure therapy on pathological fear: a randomized clinical trial
Source: Psychol Med. 2021 Jul 21;53(3):908–17. doi: 10.1017/S0033291721002270 (PMC9976021; doi:10.1017/S0033291721002270)
Supplement: Supplementary file 1 [file S0033291721002270sup001.docx]

Supplementary Table 1. Primary outcome model omnibus statistics.

| **Omnibus Statistics** | **F** | **DF** | **P-value** |
| --- | --- | --- | --- |
| Group | 2.01 | (3, 399.67) | 0.112 |
| Time | 0.6 | (1, 301.31) | 0.44 |
| Context | 2.67 | (1, 303.37) | 0.103 |
| Baseline-fear | 72.73 | (1, 371.33) | <.001 |
| Group×Time | 3.25 | (3, 301.26) | 0.022 |
| Group×Context | 3.5 | (3, 303.35) | 0.016 |
| Time×Context | 0.25 | (1, 298.32) | 0.616 |
| Group×Baseline-fear | 5.47 | (3, 370.73) | 0.001 |
| Group×Time×Context | 1.75 | (3, 298.32) | 0.157 |

Supplementary Table 2. Primary outcome model interaction contrasts.

|  | **Post-Treatment** | | | **Follow-Up** | | |
| --- | --- | --- | --- | --- | --- | --- |
| **contrast** | **estimate** | **95% CI** | **P** | **estimate** | **95% CI** | **P** |
| EX+TILS vs EX+SHAM | 2.57 | -9.46 to 14.60 | .68 | 5.68 | -6.41 to 17.77 | .36 |
| EX+TILS vs TILS | -13.3 | -25.45 to -1.15 | .03 | -4.54 | -16.77 to 7.69 | .47 |
| EX+TILS vs SHAM | -23.87 | -35.79 to -11.95 | <.001 | -13.87 | -25.98 to -1.76 | .03 |
| EX+SHAM vs TILS | -15.88 | -28.13 to -3.63 | .01 | -10.22 | -22.55 to 2.11 | .11 |
| EX+SHAM vs SHAM | -26.44 | -38.47 to -14.41 | <.001 | -19.55 | -31.78 to -7.32 | .002 |
| TILS vs SHAM | -10.57 | -22.72 to 1.58 | .09 | -9.32 | -21.67 to 3.03 | .14 |
|  | **Context-A** | | | **Context-B** | | |
| **contrast** | **estimate** | **95% CI** | **P** | **estimate** | **95% CI** | **P** |
| EX+TILS vs EX+SHAM | 1.96 | -10.11 to 14.03 | .75 | 6.3 | -5.77 to 18.37 | .31 |
| EX+TILS vs TILS | -11.47 | -23.74 to 0.8 | .07 | -6.38 | -18.53 to 5.77 | .31 |
| EX+TILS vs SHAM | -24.91 | -36.92 to -12.9 | <.001 | -12.83 | -24.88 to -0.78 | .04 |
| EX+SHAM vs TILS | -13.42 | -25.77 to -1.07 | .04 | -12.68 | -24.99 to -0.37 | .046 |
| EX+SHAM vs SHAM | -26.87 | -38.96 to -14.78 | <.001 | -19.12 | -31.33 to -6.91 | .003 |
| TILS vs SHAM | -13.44 | -25.73 to -1.15 | .03 | -6.45 | -18.74 to 5.84 | .31 |
